# Deciphering an isolated lung phenotype of NKX2-1 frameshift pathogenic variant

**DOI:** 10.3389/fped.2022.978598

**Published:** 2023-01-17

**Authors:** Céline Delestrain, Abdel Aissat, Elodie Nattes, Isabelle Gibertini, Valérie Lacroze, Stéphanie Simon, Xavier Decrouy, Alix de Becdelièvre, Pascale Fanen, Ralph Epaud

**Affiliations:** ^1^ Univ Paris Est Creteil, INSERM, IMRB, Creteil, France; ^2^ Centre Hospitalier Intercommunal de Créteil, Service de Pédiatrie Générale, Créteil, France; ^3^ Département de Génétique, AP-HP, Hopital Henri Mondor, DMU de Biologie-Pathologie, Créteil, France; ^4^ Département de Pédiatrie, Centre Hospitalier Universitaire de Tours, Tours, France; ^5^ AP-HM, Hôpital de la Conception, Service de Médecine Néonatale, Marseille, France

**Keywords:** child, NKX2-1, lung, surfactant protein, thyroid

## Abstract

**Background:**

to perform a functional analysis of a new NK2 homeobox 1 (NKX2-1) variant (c.85_86del denominated NKX2-1^DEL^) identified in a family presenting with isolated respiratory disease, in comparison to another frameshift variant (c.254dup denominated NKX2-1^DUP^) identified in a subject with classical brain-lung-thyroid syndrome.

**Methods:**

pathogenic variants were introduced into the pcDNA3-1(+)-wt-TTF1 plasmid. The proteins obtained were analyzed by western blot assay. Subcellular localization was assessed by confocal microscopy in A549 and Nthy cells. Transactivation of *SFTPA*, *SFTPB*, *SFTPC*, and *ABCA3* promoters was assessed in A549 cells. *Thyroglobulin* promoter activity was measured with the paired box gene 8 (PAX8) cofactor in Nthy cells.

**Results:**

The two sequence variants were predicted to produce aberrant proteins identical from the 86th amino acid, with deletion of their functional homeodomain, including the nuclear localization signal. However, 3D conformation prediction of the conformation prediction of the mutant protein assumed the presence of a nuclear localization signal, a bipartite sequence, confirmed by confocal microscopy showing both mutant proteins localized in the nucleus and cytoplasm. Transcriptional activity with *SFTPA, SFTPB, SFTPC, ABCA3* and *thyroglobulin* promoters was significantly decreased with both variants. However, with NKX2-1^DEL^, *thyroglobulin* transcriptional activity was maintained with the addition of PAX8.

**Conclusion:**

These results provide novel insights into understanding the molecular mechanism of phenotypes associated with *NKX2-1* pathogenic variants.

## Introduction

The transcription factor NK2 homeobox 1 (NKX2-1), a member of the NKX2 transcription factor family, partially controls the synthesis of surfactant proteins (1)*.* NKX2-1 was initially identified as a nuclear protein binding the promoter of *thyroglobulin* gene. However, various studies later showed that NKX2-1 is also expressed in lungs and brain (2). Homozygous mice lacking NKX2-1 die shortly after birth, lack lung parenchyma and the thyroid gland, and exhibit defects in the ventral region of the forebrain, including lack of a pituitary gland (3).

The human gene located in 14q13 is encoded by three exons and is characterized by its functional domain, the homeodomain in exon 3, which also contains the nuclear localization signal (NLS) (4). The most abundant and functional transcript is generated from an open reading frame starting in exon 2 and encoding 371 amino acids (5, 6). In humans, autosomal dominant NKX2-1 pathogenic variants are associated with brain-lung-thyroid syndrome (BLTS), but not always: there is a large variability in the phenotype presentation that may exclude any of the three phenotypes in BLTS (7, 8). Both neurological and pulmonary features are responsible for the disease morbidity, but lung disease is mainly responsible for the mortality (9). Various pulmonary phenotypes have been described, including respiratory distress syndrome in term infants and interstitial lung disease (ILD) in children and more recently in adults owing to abnormal lung development and the lack of pulmonary surfactant (8, 10).

Surfactant is a multi-molecular complex consisting of phospholipids (80%–90%) and proteins (10%–15%); 2% to 3% are specific proteins called surfactant protein A, B, C and D (SP-A, SP-B, SP-C and SP-D, respectively) (11). SP-B and SP-C are synthesized by alveolar type II cells as pro-peptides (pro-SP-B and pro-SP-C), then assembled with lipids into bilayer membranes and secreted into the alveolar airspace, where they form a surface film at the air–liquid interface (12, 13). Adenosine triphosphate-binding cassette transporter A3 (ABCA3) transports phospholipids from the cytoplasm into the lamellar bodies where they combine with surfactant proteins B and C (12, 13).

The thyroid involvement due to *NKX2-1* pathogenic variant is defined by thyroid dysgenesis with or without morphological abnormality of the thyroid. Various phenotypes can be observed, from congenital hypothyroidism diagnosed in the first days of life to a compensated hypothyroidism not requiring treatment (14). The NKX2-1 thyroid targets are *thyroglobulin (TG)*, *thyroperoxidase (TPO)* and *TSH receptor (TSHR)* promoters (15–17). Paired box gene 8 (PAX8) is another transcription factor involved in *thyroglobulin* promoter regulation. Functional studies showed the synergism of the association of PAX8 and NKX2-1 on the *thyroglobulin* promoter (14, 17).

The neurological symptoms associated with *NKX2-1* pathogenic variants include hypotonia in the first months of life, with motor delay and benign chorea occurring during childhood. The severity of choreic symptoms varies among families and usually stabilizes in adulthood (18). Other types of abnormal movements such as myoclonus, upper-limb dystonia and verbal tics have been described (18). In the embryonic brain, NKX2-1 controls the specification of the GABAergic interneurons and oligodendrocytes that populate the ventral and dorsal telencephalic region acting on the glial fibrillary acidic protein *(GFAP)* promoter. Loss of NKX2-1 leads to ventral to dorsal re-specification of the pallidum causing loss of GABAergic interneurons and oligodendrocytes in the dorsal telencephalic region (19). Other neurological targets of NKX2-1 have been described. NKX2-1 promotes GABAergic and cholinergic cell fate by inducing LIM homeobox 6 (LHX6) (20, 21). However, no functional test, such as luciferase assay, is available to clearly assess this function.

Here, we identified and functionally characterized two NKX2-1 variants identified in two children.

The two pathogenic variants were frameshift variants that created the same aberrant protein downstream of the 86th amino acid. The variant c.85_86del (NM_003317.3) was carried by a father and his daughter, who had isolated respiratory symptoms (8). The variant c.254dup (NM_003317.3) was identified in a boy who presented with BLTS. To understand why two frameshift pathogenic variants, produce such different phenotypes, we used functional comparative studies of both variants with thyroid- and lung-specific *in vitro* functional assays.

## Materials and methods

### Genetic analysis

After obtaining informed consent for genetic analysis according to French legislation, *NKX2-1* clinical sequencing was performed by the Genetic Department of Henri Mondor Hospital (Creteil, France). Briefly, EDTA samples were collected and used to extract leukocyte genomic DNA. Specific primers were used to amplify the coding regions and the intron–exon boundaries of *NKX2-1* (primer sequences available on request) by PCR. The PCR products were subjected to direct Sanger sequencing. For each pathogenic variant, the *de novo* or inherited status was reported.

### Plasmids

The pcDNA3-1(+)-wt-TTF1 plasmid was a gift from David Mu (Addgene plasmid #49989) (22). The plasmid was elongated by introducing an insert encompassing 131 nucleotides (nt) from the last nucleotide of exon 3 (therefore including the stop codon created by the frameshift variant). Then the variants were introduced by site-directed mutagenesis (Stratagene, Waghaeusel-Wiesental, Germany). For transactivation experiments, we used the following plasmids: reporter gene containing NKX2-1 and PAX8 binding sites from the *thyroglobulin* promoter upstream of luciferase (19), SP-B promoter luciferase (23) for surfactant protein B (*SFTPB*) promoter, pGL2-SP-C-promoter (24) for surfactant protein C (*SFTPC*) promoter, pGL3-ABCA3-promoter (25) for *ABCA3* promoter, pGL3-SP-A2-promoter (26) for surfactant protein A (*SFTPA*) promoter, pcDNA3.1-PAX8 (27), and hGFAP-fLuc [gift from Albee Messing, Addgene plasmid # 40589, (28)].

### Cell culture

Human lung epithelial cells, A549 cells (ATCC, CCL-185, Manassas, VA), were cultured in DMEM-glutamax (Thermo Fisher Scientific, Waltham, MA) with penicillin-streptomycin (Thermo Fisher Scientific) and 10% FCS (Eurobio, Courtaboeuf, France). Immortalized human thyroid cell lines, Nthy-ori3.1 cells [gift from A. Carré and M. Polak, (29)], were cultured in RPMI-Glutamax (Thermo Fisher Scientific, Waltham, MA) with penicillin-streptomycin (Thermo Fisher Scientific) and 10% FCS (Eurobio, Courtaboeuf, France).

### In vitro synthesis of NKX2-1

Proteins were synthesized from the wild-type and mutated *NKX2-1* plasmids (1 µg) by *in vitro* transcription/translation with the TNT-T7–coupled reticulocyte lysate system (Promega, Madison, WI, USA). Western blot analysis was performed with 1 µl TNT reaction as explained below.

### Western blot assay

In total, 6.5 × 10^5^ A549 and 1.1 × 10^6^ Nthy cells were plated in 60-mm-diameter culture plates. Cells were transiently transfected with 3 µg wild-type or mutated *NKX2-1* by means of lipofectamine 3,000 (Thermo Fisher Scientific) according to the manufacturer's instructions. At 24 h after transfection, proteins were extracted with RIPA lysis and extraction buffer (Thermo Fisher Scientific) and quantified by using Bio-Rad DC protein assay (BioRad, Hercules, CA, USA). A 30-µg amount of protein was fractionated by SDS-PAGE on 12% tris-glycine gels. Proteins were further electrotransferred to a PVDSF 0.45-µM membrane (GE Healthcare, Little Chalfont, UK). Blots were probed overnight at 4°C with NKX2-1 antibody (Abgent, San Diego, CA, USA, 1/500), then incubated with secondary antibody. Bound antibodies were detected by using ECL prime chemiluminescence substrate (Amersham ECL, GE Healthcare) and exposed to a G:Box (Syngene, Cambridge, UK). Images were analyzed by using GeneSys software (Daly City, CA, USA). Experiments were repeated three times.

### Immunocytofluorescence

For immunostaining, 2.6 × 10^5^ A549 and 4.5 × 10^5^ Nthy cells per well were grown on coverslips in 6-well plates for 24 h. Then cells were transiently transfected with 2 µg wild-type or mutated *NKX2-1* by means of lipofectamine 3,000 (Thermo Fisher Scientific). At 24 h after transfection, cells were washed and fixed with acetone/methanol (3/7) for 5 min at 4 °C and blocked with anti-NKX2-1 antibody (1:250 dilution; Abgent) and Alexa fluor 488 rabbit secondary antibody (1:2000 dilution; Molecular Probes, Eugene, OR, USA). Fluorescence was visualized with a Zeiss confocal microscope. Experiments were repeated three to four times.

### Transactivation

For luciferase assay, 1.2 × 10^5^ A549 and 10^5^ Nthy cells per well were plated in 24-well plates. After 24 h, A549 cells were transfected with 400 ng lung reporter plasmid (SP-A2, SP-B, SP-C, or ABCA3 luciferase), and 400 ng wild-type or mutant NKX2-1 plasmid. Nthy cells were transfected with 200 ng *thyroglobulin* reporter plasmid, 200 ng PAX8 plasmid and 200 ng wild-type or mutant NKX2-1 plasmid. The total amount of plasmids was kept constant by the addition of the respective empty vector (pcDNA3-1). After 24 h, cells were harvested, and underwent firefly luciferase assay (Promega) in a TriStar system (Berthold Technologies GmbH & Co. KG, Germany). The promoter activation was calculated after normalization to the total luminescence of the experiment, in five independent experiments, each condition performed in duplicate.

### Statistical analysis and in silico analysis

One-way ANOVA (GraphPad Prism 6, GraphPad Software, La Jolla, CA, USA) was used to analyze differences in transactivation activities. Data are presented as mean ± SEM. The mutated proteins were analyzed by using InterPro (30) and PROSITE (31) software to find new domains and with the cNLS mapper bioinformatic program to find the NLS (32). Multiple sequence alignment was analyzed by using the Clustal Omega online tool (https://www.ebi.ac.uk/Tools/msa/clustalo/) (33).

## Results

### Subjects

The first index subject was an infant girl born at 38 weeks from a healthy nonconsanguineous couple. At 7 months, she showed failure to thrive and tachypnoea. High-resolution CT (HRCT) revealed diffuse ground-glass opacities suggesting ILD. At 10 months, she required supplemental oxygen support. She received azithromycin, hydroxychloroquine, and corticosteroid pulses. Corticosteroid treatment was stopped at age 3 years (after 27 pulses) after HRCT-evidence of improvement, but she required nocturnal oxygen supplementation until age 4 years. Thyroid function and morphology were within the normal range and remained stable for 8 years' follow up (TSH level = 4 (7 months), 4.9 (2 years 6 months), 3.2 (4 years 7 months) and 3.6 mUI/L (7 years 4 months) for a normal value < 5 mUI/L). During this time, the girl showed normal psychomotor development (with regard to a general pattern of physical, emotional, intellectual and social milestones) with absence of hypotonia, abnormal movements, or motor coordination (gross or fine) abnormalities. She received enteral nutrition by gastrostomy from age 15 months for 6 years. The genetic diagnosis was achieved at 2 years. Her father carried the same variant and had a diagnosis of ILD later in life (age 30 years) during an intensive-care-unit hospitalization for severe viral respiratory infection. He also had normal thyroid function and absence of neurological symptoms. His last HRCT revealed a slight progression of certain fibrotic zones. Finally, her grandmother died without genetic analysis of pulmonary fibrosis complicated by lung cancer.

The second index subject was a term-born boy showing respiratory distress syndrome within the first day of life. A chest radiograph showed diffuse ground-glass opacities, compatible with severe hyaline membrane disease. After the first week of life, the boy showed good respiratory condition, with no evidence of respiratory distress recurrence, and a 6-min walk test performed at age 4 years gave normal results. Neonatal screening revealed congenital hypothyroidism [TSH level 420 mU/L and free thyroxine (T4) level 0.008 nmol/L for a normal value < 0,6 mU/L and from 120 to 210 nmol/L before the first week for TSH and free thyroxine respectively], and levothyroxine was started on day 7. Scintigraphy revealed thyroid ectopia with sublingual localization. The boy showed hypotonia at age 3 months and psychomotor delay, with autonomous walking achieved at age 2 years. The choreoathetotic movements appeared at age 3 years.

### Identification and characterization of NKX2-1 human variant

**Figure 1:**
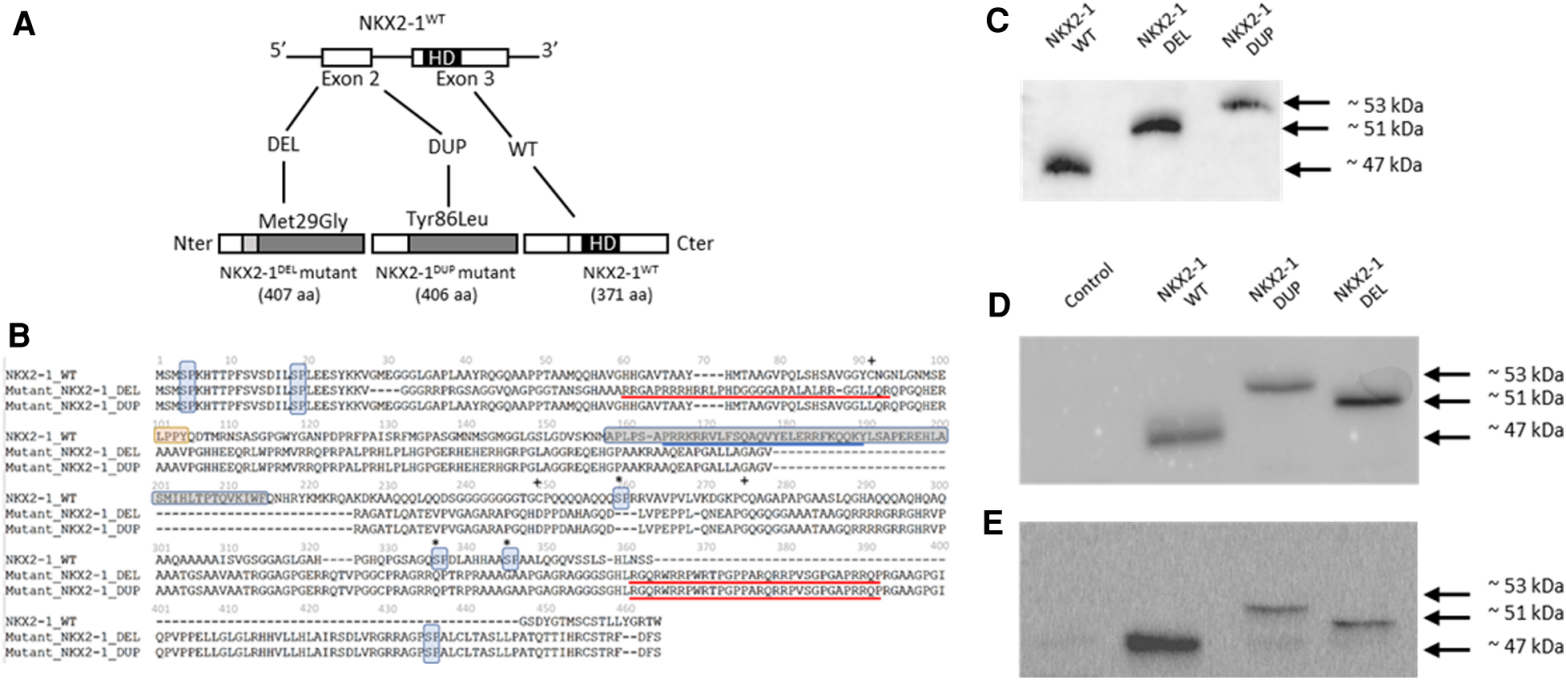
(**A**) Identification of two NKX2-1 pathogenic variant by direct sequencing and expression of the wild-type and mutated NKX2-1 protein. The diagram of the NKX2-1 transcript variant 2 (NM_003317.3) shows the position of the homeodomain and variants. (**B**) Deduced amino acid sequences of NKX2-1^dup^ and NKX2-1^del^ as compared with NKX2-1^WT^. The nuclear localization signal (NLS) is underlined in blue for the NKX2-1^WT^ and the putative NLSs, according to the predictions, are underlined in red for the two mutants. The homeobox domain is framed in gray (position 160 to 220) for the NKX2-1^WT^ and is absent for both mutants. pSP motifs have been highlighted in light blue. The NKX2-1^WT^ harbors five motifs while NKX2-1^del^ and NKX2-1^dup^ have three motifs. LPPY motif, a putative TAZ protein interaction motif, has been highlighted in orange for the NKX2-1-^WT^. This motif is absent for both mutants. Serines involved in phosphorylation in the wild-type sequence but affected by the mutations are marked by an asterisk. Cysteines involved in the dimerization of the amino-acid chains in the wild-type but affected by the mutations are marked by a plus sign (adapted from Moya et al., 2018). (**C**) Representative western blot analysis in pcDNA3-1(+)-TTF1 plasmid of translated NKX2-1 wild type and mutant (DEL and DUP). (**D**) Representative immunoblotting of NKX2-1 in whole cell lysates of A549 cells not transfected (Control) or transiently transfected with NKX2-1^WT^, NKX2-1^DUP^ and NKX2-1^DEL^. (**E**) Representative immunoblotting of NKX2-1 in whole cell lysates of Nthy cells non-transfected (Control) or transiently transfected with NKX2-1^WT^, NKX2-1^DUP^ and NKX2-1^DEL^.

For both subjects, screening for surfactant gene *SFTPC*, *SFTPB* and *ABCA3* was negative by direct sequencing of the subject's DNA. However, we identified an inherited heterozygous variant in *NKX2-1* for the girl. The pathogenic variant was a deletion of an adenosine and a thymidine at positions 85 and 86 of the cDNA (transcript NM_003317.3), which generates a frameshift leading to an aberrantly longer protein of 407 amino acids (NKX2-1^DEL^), retaining only the first 28 amino acids of the wild type (Figure 1A).

We identified a *de novo* heterozygous variant in the *NKX2-1* gene for the boy. The variant was a duplication of guanine at position 254 of the cDNA (transcript NM_003317.3), which generates a frameshift leading to an aberrantly long protein of 406 amino acids (NKX2-^DUP)^), retaining only the first 85 amino acids of the wild type (Figure 1A).

Amino acid prediction of the mutants by bioinformatic tools anticipated a severe impact on protein function based on reduced conservation of the amino acid sequence with respect to the wild-type protein and the absence of critical domains of this transcription factor, i.e, the homeodomain and the native NLS. Both mutants have lost several serine-proline (SP) residues in the second half of the amino-acid sequence. They also have lost several cysteines which could be involved in dimerization of the NKX2-1 protein (Figure 1B).

### Protein expression and subcellular localization of NKX2-1 mutants

**Figure 2:**
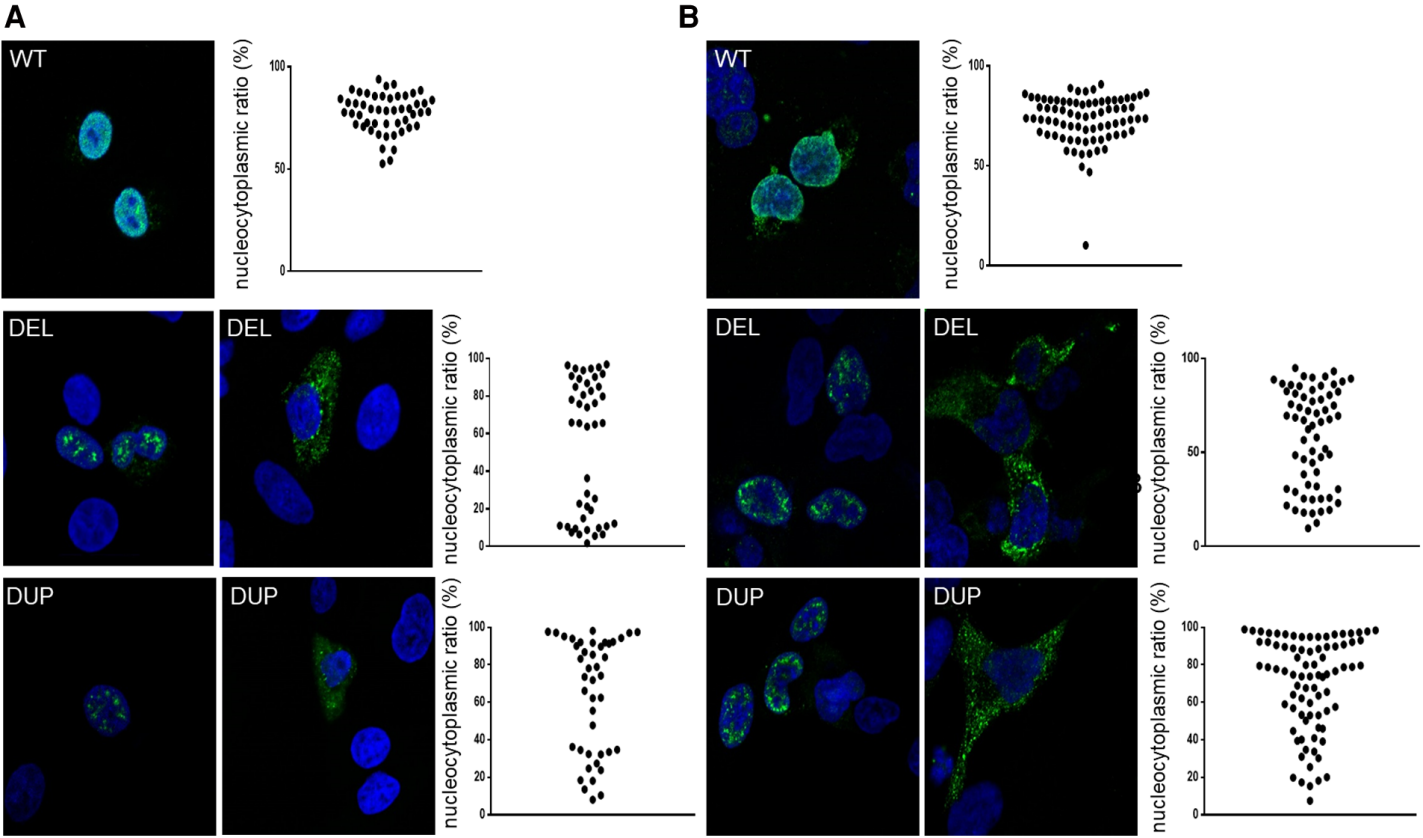
Representative confocal images of A549 cells (**A**) and nthy cells (**B**) after 24-h expression of NKX2-1^WT^, NKX2-1^DEL^ and NKX2-1^DUP^. Immunostaining involved incubation with rabbit polyclonal anti-NKX2-1 antibody (green). Nuclei were detected by ToPro-3 iodide (blue). Scatter graphs show the nucleocytoplasmic ratio of each protein (≥10 cells per experiment, *n* = 3 or 4).

To assess the functional consequences of these two pathogenic variants, we used an *in vitro* transcription and translation system to synthesize the wild-type and NKX2-1^DUP^ and NKX2-1^DEL^ proteins; the identity of the sequences obtained were verified by mass spectrometry (data not shown). As expected, the two mutated proteins had higher molecular weight than the wild type (Figures 1C–E). *In silico* analysis using a cNLS mapper bioinformatics program, predicted the absence of consensus nuclear localization signal in the two mutants. Considering the 3D conformation of the protein, it also showed the presence of an NLS bipartite that may explain the nuclear localization of both mutants (Supplementary Figure S1). To confirm these predictions, we then performed confocal microscopy analysis of A549 (Figure 2A) and Nthy cells (Figure 2B) transfected with both mutants and the wild-type NKX2-1. As expected with the loss of the NLS, both mutant proteins were localized in cytoplasm, but as predicted by the identification of putative bipartite NLSs, both mutants were also localized in the nucleus. However, they exhibited a very different pattern of expression in the nucleus as compared with the wild-type protein, forming aggregates in the nucleus in each cell line.

### Transcriptional activity of NKX2-1^wt^, NKX2-1^DUP^ and NKX2-1^DEL^ on tissue-specific promoters

**Figure 3:**
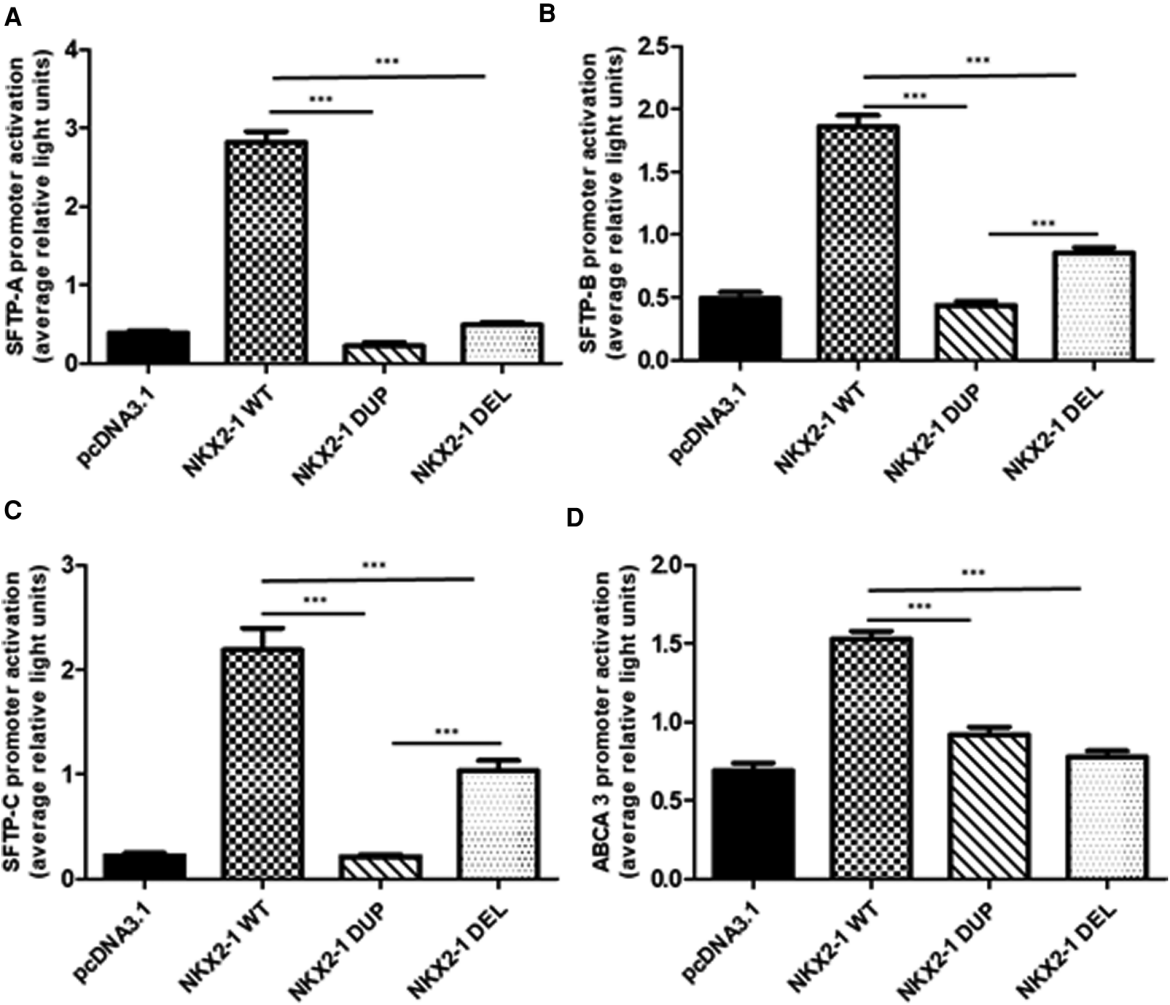
Functional activity of wild-type vs. mutant NKX2-1 on promoters of lung-specific genes (*SFTPC*, *SFTPB*, *ABCA3* and *SFTPA*). Transactivation of *SFTPA* (**A**), *SFTPB* (**B**), *SFTPC* (**C**), and *ABCA3* (**D**) by wild-type NKX2-1, NKX2-1^DUP^ or NKX2-1^DEL^. Promoter constructs were co-transfected in A549 cells (**A–D**) with the empty pcDNA3.1 vector or expression vector for the wild-type or mutated NKX2-1 cDNAs. Data are mean ± SEM of five independent experiments performed in duplicate. *p < 0.05, **p < 0.01, ***p < 0.001.

**Figure 4:**
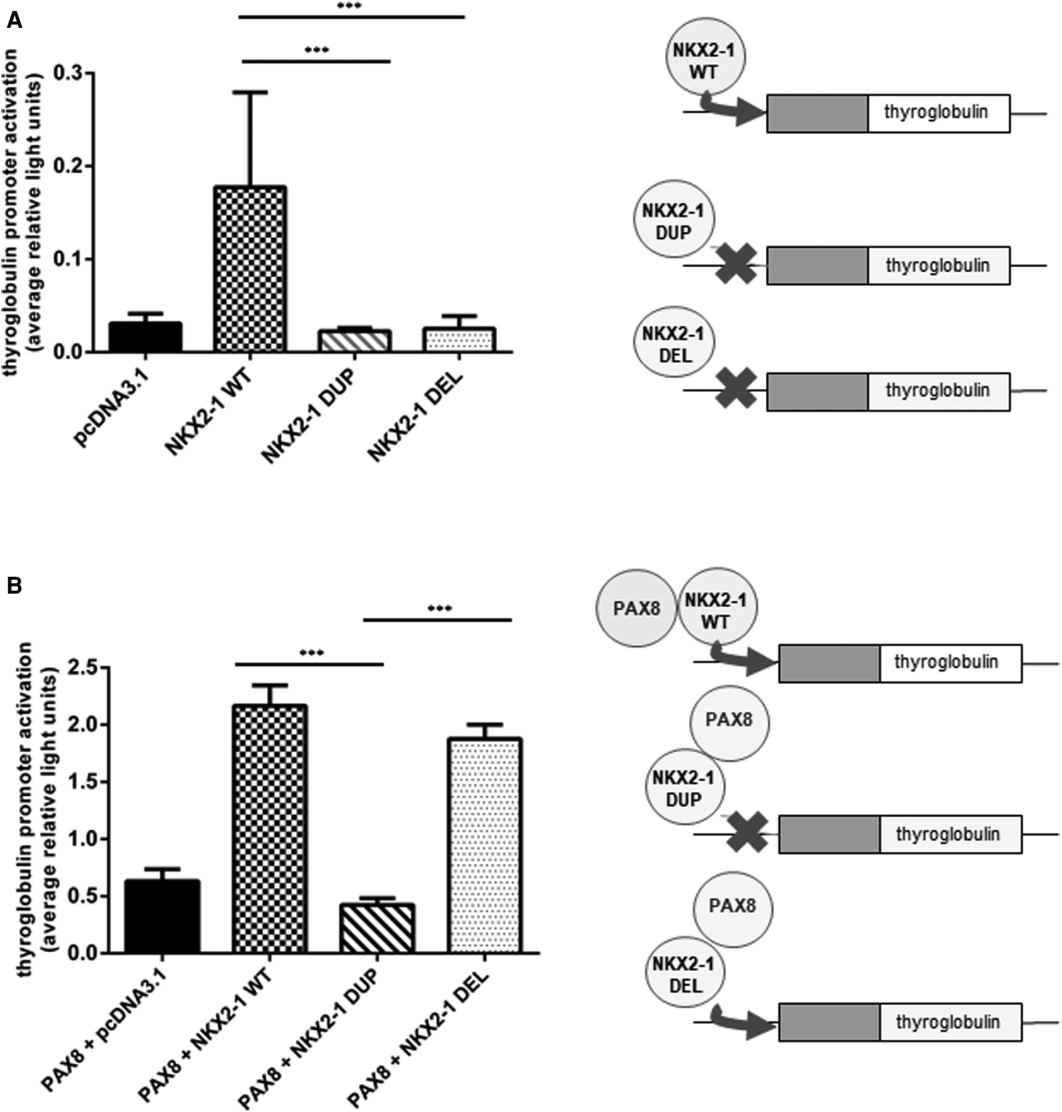
Functional activity of wild-type vs. mutants NKX2-1 on thyroid-specific (thyroglobulin) gene. Transactivation of the thyroglobulin promoter by NKX2-1^WT^, NKX2-1^DUP^ or NKX2-1^DEL^. Promoter constructs were co-transfected without (**A**) or with (**B**) PAX8 in Nthy cells with the empty pcDNA3.1 vector or the expression vector for wild-type or mutated NKX2-1 cDNAs. Data are mean ± SEM of five independent experiments performed in duplicate. ***p < 0.001.

Given the nuclear localization of the two mutants, we then aimed to study their transcriptional activity. We measured the transcriptional activity of the mutant proteins by using representative target promoters specific for the tissues affected in BLTS: *SFTPA*, *SFTPB*, *SFTPC*, and *ABCA3* for lung and *thyroglobulin* for thyroid. As compared with the wild-type protein, the two mutant proteins added to the four lung and *thyroglobulin* promoters showed significantly decreased activity (Figure 3). Transcriptional activity of *thyroglobulin* promoter was also assessed with the addition of the co-factor PAX8. *Thyroglobulin* promoter activation was significantly decreased by NKX2-1^DUP^ as compared with the wild type (Figure 4). In contrast, in agreement with the clinical phenotype and despite the absence of homeodomain, *thyroglobulin* promoter activity was significantly increased by NKX2-1^DEL^, similar to the wild type (Figure 4).

## Discussion

In the present report, we describe two heterozygous pathogenic variants in the *NKX2-1* transcription factor. The first frameshift pathogenic variant, c.85_86del, was carried by a girl who showed ILD during the first year of life. This girl and her father, who carried the same variant, exhibited only lung disease. The second variant, c.254dup, previously described (19), was carried by a boy who had BLTS. The boy had respiratory distress syndrome at birth, but the condition improved progressively without treatment. Congenital hypothyroidism was diagnosed at birth, and the boy also showed choreic movements and psychomotor delay.

These two variants shift the reading frame in the same way after the 86th amino acid, leading to aberrant proteins associated with different BLTS phenotypes. Variants (*n* = 10) that shift the reading frame in the same way leading to an aberrant protein of 406 amino acids with deletion of the functional homeodomain were previously described (10, 34–36). Seven of the 9 subjects described had BLTS; two of them had thyroid and neurological symptoms, and one presented thyroid and respiratory symptoms.

To improve our understanding of the molecular basis of this heterogenous phenotype, we first performed an *in vitro* study to compare the NKX2-1^DUP^ and NKX2-1^DEL^ mutant proteins. Indeed, western blots showed that both had higher molecular weight than NKX2-1^WT^, the weight of NKX2-1^DUP^ being much higher than NKX2-1^DEL^ in both Nthy and A549 cells. These discrepancies could be explained by the functional motifs within the amino acid sequence of the NKX2-1^wt^ protein that were affected by the pathogenic variants (Figure 1B). NKX2-1 posttranslational modifications, such as phosphorylations, acetylations, and redox states could alter their molecular weight, mobility in electrophoresis assays and transcriptional activity (37). Among the five SP motifs present in the NKX2-1^wt^ protein, three were absent in mutants, these latter being important in the phosphorylation state of the protein and its cellular-specific transcriptional activity (38). One putative new SP motif appeared at the Cter of both mutants and could also impact their phosphorylation state and thus their activity. Additionally, three cysteines involved in the dimerization of the wild-type protein (39) disappeared in both mutants, whereas four other cysteines appeared which could disturb the dimerization of the mutants and/or their redox state (40), and consequently affect the molecular weight. We also showed that despite the loss of the native NLS, the two mutant proteins could reach the nucleus, according to putative NLSs that could be present in both mutant sequences (Supplementary Figure S1). Indeed, in both cell lines, the mutants were in cytoplasm but also in the nucleus, where they formed aggregates instead of the diffuse appearance seen with the wild-type protein. The cell nucleus contains distinct classes of subnuclear structures (cajal bodies, splicing speckles, paraspeckles). Many nuclear proteins are known to interact dynamically with one or another of these bodies, and disruption of the specific organization of nuclear proteins can result in defects in cell functions and may cause molecular disease (41). Further experiments to explain the subnuclear re-localization of the two mutants in one or several of these specific nuclear structures are needed and would help refine the cartography of functional domains within the NKX2-1 protein sequence.

Because the mutant proteins can reach the nucleus, they could activate tissue-specific promoters such as *thyroglobulin*, a specific thyroid gene. Of note, unlike NKX2-1^DUP^, NKX2-1^DEL^ was able to transactivate *thyroglobulin* but required a PAX8 cofactor for its activity. PAX8 and NKX2-1 are both expressed only in the thyroid, and PAX8 has a synergic effect with NKX2-1 to transactivate the *thyroglobulin* promoter (14, 17). Despite its highly modified amino acid, NKX2-1^DEL^ protein could transactivate the *thyroglobulin* promoter with PAX8, which suggests that PAX8 is essential for the thyrocyte-specific promoter activation of *thyroglobulin* (42–44). Both mutants have lost their homeodomain and have equivalent sequence sizes. However, they differ significantly from amino-acids 30 to 88 in their sequences, suggesting that this part of the Nterm region could be of interest for a selective binding of the PAX8 cofactor and putative other factors, such as TAZ. Although the two mutants we studied have both lost the LPPY motif (illustrated in Figure 1B), which may be involved in the binding of TAZ protein (45), a mechanism involving both TAZ and PAX8 within this Nterm region of NKX2-1 could be possible. Interestingly, Moya et al. (36) showed that the TAZ cofactor could bind to the 74 first amino-acids of the NKX2-1 protein, maybe through pS-P in the Nterm region. Thus, further experiments focusing on binding of both TAZ and PAX8 on the 30-to-74 first amino acids of NKX2-1 could help to decipher their roles in the tissue-specificity of NKX2-1. Synergy with PAX8 can overcome the functional defect of a specific NKX2-1 variant on the *thyroglobuli*n promoter (14). PAX8 may recruit NKX2-1 *via* protein–protein interaction without NKX2-1 binding to DNA (46, 47). For the present variants, we hypothesize that the loss of the NKX2-1^DEL^ homeodomain was associated with the creation of a new interaction domain with PAX8, thus allowing for *thyroglobulin* promoter transactivation.

To gain insight into the mechanism of the pulmonary phenotype of our two subjects, we assessed transactivation of specific surfactant gene promoters, including *SFTPA*, *SFTPB*, *SFTPC* and *ABCA3*, in two cell lines. All these proteins are essential for surfactant homeostasis and normal lung development (48–53). Both mutant proteins resulted in decreased luciferase activity suggesting reduced transcriptional activity of the *SFTPA*, *SFTPB*, *SFTPC* and *ABCA3* promoters, which is consistent with the pulmonary phenotype of our two subjects. In the 2 index children, the lung phenotype differed as one child presented with severe respiratory distress with rapid improvement with the first weeks, while the other experienced a progressive respiratory failure at 7 months evolving to chILD. Moya et al. have studied 2 children with BLTS, but one with absence of lung involvement. The authors demonstrated that the coactivator TAZ/WWTR1 (transcriptional coactivator with PDZ-binding motif/WW domain-containing transcription regulator protein (1) was able to restore NKX2-1 transactivation suggesting a potential role of TAZ in the development of the lung disease (36). Alternatively, we and others have shown that decreased surfactant protein levels may be associated with a less severe phenotype as compared with complete deficiency (54). The distinct respiratory phenotypes (chILD vs. resolved neonatal RDS) might be related to the differences in the level transcription of SP-B and SP-C which were significantly decreased in NKX2-1^DUP^ compared to NKX2-1^DEL^. Indeed SP-B and SP-C protein levels are crucial in the adaptation of the newborn at extrauterine conditions (55) as illustrated by SP-B deficiency causing severe neonatal respiratory distress and death within the first few months of life (56) whereas chILD is mainly triggered by surfactant metabolism disruption due to abnormal proSP-C protein together with decrease ABCA3, SP-C and SP-B levels (57).

In conclusion, here we explain the difference in phenotype in two subjects carrying two frameshifts *NKX2-1* pathogenic variants, c.85_86del and c.254dup. The two variants (dup + 1nt/del-2nt) produce an aberrant protein of 407 and 406 amino acids identical from the 86th amino acid lacking the functional homeodomain and the nuclear localization signal. Unexpectedly, 3D conformation prediction of the mutant protein assumed the presence of a nuclear localization signal, a bipartite sequence, which was experimentally confirmed by confocal microscopy showing both mutant proteins localized in the nucleus and cytoplasm. However, only the c.175_176del NKX2-1 mutant could transactivate the *thyroglobulin* promoter because of the PAX8 cofactor. Other functional studies are warranted to further elucidate the molecular mechanisms involved in *NKX2-1* variants associated with heterogeneous phenotypes.

## Data Availability

The original contributions presented in the study are publicly available. This data can be found here: 10.6084/m9.figshare.21898911.
