# Supplementary material for: Deciphering an isolated lung phenotype of NKX2-1 frameshift pathogenic variant
Source: Front Pediatr. 2023 Jan 17;10:978598. doi: 10.3389/fped.2022.978598 (PMC9888430; doi:10.3389/fped.2022.978598)
Supplement: Supplementary file 1 [file Datasheet1.pdf]

A

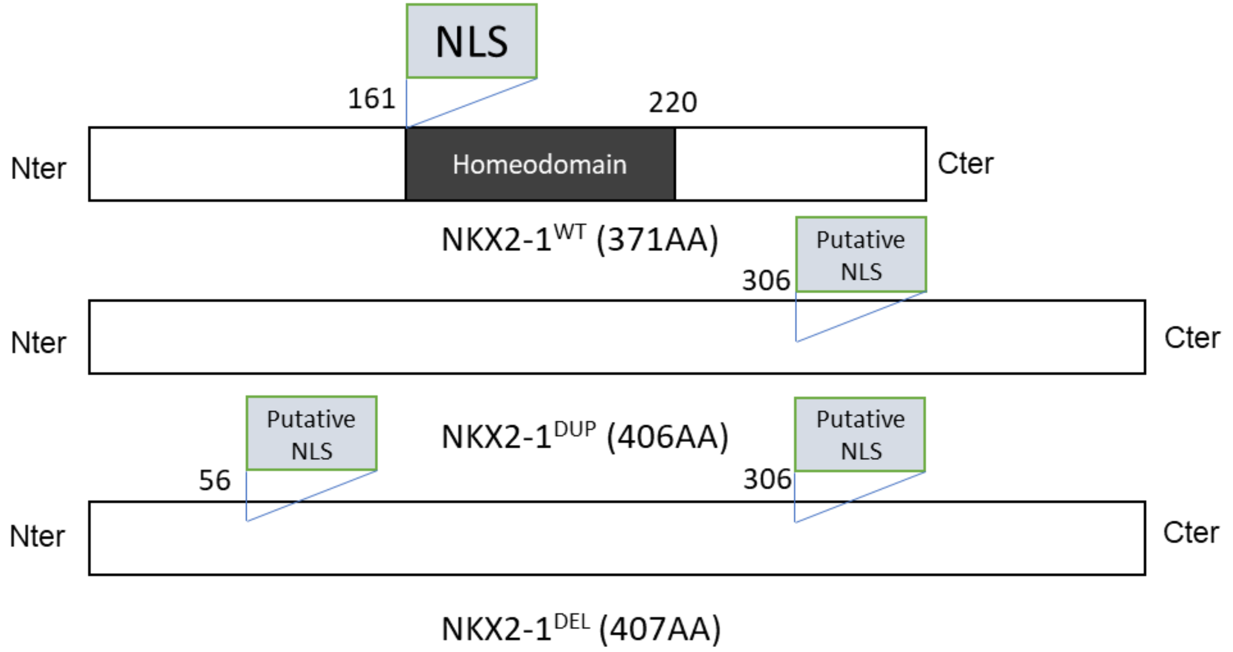

B

Predicted monopartite NLS		
Pos.	Sequence	Score
160	PRRKRRVLF	4.5
160	PRRKRRVLFS	13

Predicted bipartite NLS		
Pos.	Sequence	Score
161	RRKRRVLFSQAQVYELERRFKQQKY	6.1
160	PRRKRRVLFSQAQVYELERRFKQQKYLSAP	4.9
160	PRRKRRVLFSQAQVYELERRFKQQKY	10.6
160	PRRKRRVLFSQAQVYELERRFKQQKYLSAP	7.8
160	PRRKRRVLFSQAQVYELERRFKQQKYLSAPE	4.7
161	RRKRRVLFSQAQVYELERRFKQQKY	4.6
161	RRKRRVLFSQAQVYELERRFKQQKYLSAP	4.5
178	RRFKQQKYLSAPEREHLASMIHLTPTQVKIWF	4.6

NKX2-1^{WT} (371AA)

Predicted monopartite NLS		
Pos.	Sequence	Score

Predicted bipartite NLS		
Pos.	Sequence	Score
56	RRGAPRRRHRRLPHDGGGGAPALALRRGGLLQR	4.4
56	RRGAPRRRHRRLPHDGGGGAPALALRRGGLLQRQP	4.3
305	RGQRWRRPWRTPGPPARQRRPVSGPGAPRRQP	4.1

NKX2-1^{DEL} (407AA)

Predicted monopartite NLS		
Pos.	Sequence	Score

Predicted bipartite NLS		
Pos.	Sequence	Score
306	RGQRWRRPWRTPGPPARQRRPVSGPGAPRRQP	4.1

NKX2-1^{DUP} (406AA)

Supplemental Figure 1. Prediction of subcellular localization of NKX2-1^{WT} and mutants NKX2-1^{DUP} et NKX2-1^{DEL}. (a) Schematic representation of wild-type protein and mutants with the localization of the nuclear localization signal (NLS) for WT and the putative NLS for the mutants. (b) Prediction of localization of proteins with bipartite NLS by using NLS mapper (http://nls-mapper.iab.keio.ac.jp/cgi-bin/NLS_Mapper_form.cgi). Score > 7: nucleus. Score 7: partially in nucleus. Score 2-6: nucleus and cytoplasmic. Score <3: cytoplasmic. The cut-off score has been set at 4.0.
